# The Role of Social Media Interaction in Developing Intercultural Digital Communication Competence: A Systematic Literature Review

**DOI:** 10.3390/bs16050794

**Published:** 2026-05-16

**Authors:** Chenxi Zhang, Salina Husain, Roslina Mamat

**Affiliations:** Faculty of Modern Languages and Communication, Universiti Putra Malaysia, Seri Kembangan 43400, Selangor, Malaysia; gs64649@student.upm.edu.my (C.Z.);

**Keywords:** intercultural digital communication, social media interaction, cross-cultural pragmatics, computer-mediated communication, higher education

## Abstract

As higher education becomes more digitally mediated and internationally oriented, social media interaction has increasingly become a crucial way across linguistic and cultural boundaries. It functions as a space where interaction unfolds, and meanings are negotiated. To explore how it contributes to the development of intercultural digital communication competence, this study conducted a systematic literature review. Following a PRISMA-guided process, we identified 19 empirical studies published between 2015 and 2025 and evaluated them using the Mixed Methods Appraisal Tool (MMAT). The findings suggested that this competence develops through the combined influence of pragmatic resources, ongoing behavioral adjustment, and affective factors. Features of politeness strategies, stance-taking, and multimodal cues play a noticeable role in shaping interaction. The result showed that participation alone does not automatically lead to improvement, and structured support and opportunities for reflection also make a difference. These findings offered implications for communication training and digitally mediated learning in higher education.

## 1. Introduction

With the trend toward greater internationalization and digitalization in higher education, students are increasingly called upon to interact across cultures within digital spaces. These interactions revolve around both linguistic and behavioral actions, involving how individuals interpret social cues, manage interpersonal relationships, and make moment-to-moment communicative decisions in computer-mediated contexts (Garcia et al., 2019). In many Sciences, Technology, Engineering, and Mathematics (STEM) programs, students work in project teams composed of members from different countries and rely on digital tools such as online laboratories, virtual design reviews, and communication platforms to coordinate their work (Blyth, 2018). These interactions take place in contexts characterized by cultural diversity, where differences in communication styles, expectations, and interpretations can influence how meaning is constructed and understood.

Within these environments, communication is not only a linguistic activity but also a form of social behavior. In computer-mediated communication (CMC), students continuously interpret cues, make decisions about how to respond, and adjust their communicative behavior in relation to others. This involves sensitivity to cultural differences, the ability to manage interpersonal relationships, and an awareness of how meaning is negotiated in interaction (Koivumäki & Wilkinson, 2022). Rather than simply transmitting information, students engage in ongoing processes of interpretation, evaluation, and adaptation.

In response to these changing conditions, researchers in higher education and the learning sciences have increasingly conceptualized intercultural digital communication competence as a key capability for students. Early models of intercultural communicative competence provide a useful foundation (Deardorff, 2006), but more recent work suggests that competence in digital environments involves additional behavioral dimensions. These include the ability to regulate communicative choices in online interaction, to interpret culturally variable cues, and to respond appropriately within digitally mediated contexts (Rajagopal et al., 2020; Wongsuban et al., 2024). From this perspective, intercultural digital communication competence is defined as the capacity to interpret, negotiate, and adapt communicative behavior in digitally mediated intercultural interactions. This competence involves the use of pragmatic and multimodal resources, sensitivity to cultural and contextual differences, and the capability to adjust responses for audience expectations. It is conceptualized as an adaptive and interactionally situated form of communicative behavior that develops through participation in digital communication environments. This definition draws on prior work in intercultural communicative competence and digital pragmatics, which emphasizes the role of contextual sensitivity, interactional adaptation, and meaning negotiation (Smith, 2024).

Social media platforms and other forms of computer-based communication now play an increasingly prominent role in students’ everyday academic and collaborative activities. Students use messaging applications and social networking sites to coordinate tasks, negotiate roles, and maintain relationships within project teams. These environments function as informal learning spaces, where communication practices develop through participation rather than direct instruction (Qi, 2018). The behavioral patterns that emerge in these spaces include how students manage disagreement, express agreement, or maintain engagement. All of these can have direct consequences for teamwork, conflict resolution, and knowledge sharing.

From a behavioral perspective, cross-cultural pragmatics offers a useful lens for examining how these interactional processes unfold. It focuses on how individuals make context-sensitive communicative choices, including how they express politeness, manage face concerns, and align with others in interaction. These choices are often subtle but consequential, influencing both relational outcomes and task-related collaboration. Research in pragmatics and CMC has shown that such behaviors vary across cultural contexts, particularly in the use of speech acts, politeness strategies, and facework in online communication (Bardis et al., 2021; Burgess et al., 2021; Handford et al., 2019).

The increasing use of social media further complicates these processes by introducing multimodal forms of communication. Emojis, reactions, and visual elements serve as additional behavioral cues that shape how meaning, stance, and emotion are expressed and interpreted (Chen & Feng, 2023; Fatima et al., 2025). While these resources can support relational work, they can also introduce ambiguity, particularly in intercultural contexts where interpretations of such cues may differ.

Although research on cross-cultural pragmatic behavior in social media has grown, it remains fragmented across disciplines, including applied linguistics, communication studies, and educational technology (Al-Rahmi et al., 2022; Greenhow et al., 2020; Liu et al., 2022; Shobirin et al., 2018). Many studies focus on isolated features, such as specific speech acts or interactional strategies, without fully accounting for how these behaviors interact within broader communication processes. At the same time, research on teamwork and communication in higher education often treats intercultural competence at a general level, without examining the micro-level behavioral patterns that shape everyday interaction. As a result, there is still limited understanding of how social media-based communication supports the development of intercultural digital communication competence.

This lack of synthesis also creates practical challenges for educators. Without a clearer understanding of how students actually communicate and behave in online interaction, it becomes difficult to design effective support for communication, address misunderstandings, or foster inclusive collaboration (Basantes-Andrade et al., 2025). Existing reviews tend to focus either on technological tools or on face-to-face communication, leaving digitally mediated interaction relatively underexplored.

To fill these gaps, the present study conducts a systematic literature review of empirical research on cross-cultural pragmatics in social media-based communication among higher education students. The review aims to map research trends and methodological approaches, identify key behavioral and pragmatic features that shape intercultural digital communication, and examine how these processes relate to competence development in digitally mediated learning environments. By bringing together insights from pragmatics and the learning sciences, the study seeks to contribute to a more behaviorally grounded understanding of digital communication in higher education. The following research questions are therefore proposed to guide the analysis.

RQ1: How have researchers examined cross-cultural pragmatics in social media-based communication among higher education students?

RQ2: What pragmatic and interactional features are associated with intercultural digital communication competence in social media contexts?

RQ3: How do technology-supported teaching designs contribute to the development of intercultural digital communication competence in higher education?

These research questions provide the analytical framework for interpreting the reviewed studies. RQ1 focuses on how cross-cultural pragmatics has been investigated in prior research, including the settings examined, participant groups, digital platforms, and methodological approaches adopted. RQ2 addresses the pragmatic and interactional resources linked to intercultural digital communication competence, such as speech acts, stance-taking, politeness practices, and multimodal elements, including emojis. RQ3 considers the role of technology-supported pedagogical designs in fostering this competence.

## 2. Theoretical Framework

This study draws on perspectives from cross-cultural pragmatics, intercultural communication, and behavioral approaches to digitally mediated interaction to examine how intercultural digital communication competence is enacted in social media-based environments. Normally, traditional approaches treat communication as a set of stable linguistic forms (Benz et al., 2011). However, this framework conceptualizes it as a form of adaptive behavior that emerges through interaction and is shaped by contextual, cognitive, and affective processes.

From a pragmatic perspective, communication involves the strategic use of language and other semiotic resources to manage meaning and social relationships. Politeness theory (Brown & Levinson, 1987) provides a useful foundation by framing communicative choices as forms of face management. In intercultural contexts, these strategies are not universally shared but are shaped by culturally specific norms and expectations (Kimura, 2024). As a result, communication becomes an interpretive process in which individuals must continuously adjust their behavior in response to how their messages are perceived by others. This perspective directly informs RQ2, which focuses on the pragmatic and interactional features associated with intercultural digital communication competence.

At the same time, communication in social media environments can be understood as a form of behavioral adaptation within computer-mediated contexts. Compared with face-to-face interaction, digital communication often involves reduced contextual cues and increased reliance on multimodal signals such as emojis, text formatting, and timing of responses (Lu et al., 2025). Therefore, Individuals should interpret partial or ambiguous information and make context-sensitive decisions about how to respond. These processes involve cognitive dimensions, such as attention and interpretation, as well as affective dimensions, including confidence, uncertainty, and engagement. It has been suggested that communicative behavior is not fixed but dynamically adjusted through interaction (Malyndra et al., 2020; Rajan, 2025). This perspective provides a conceptual basis for RQ1, which examines how researchers have studied such behavior across different digital platforms, interactional settings, and methodological approaches.

In addition, insights from the learning sciences and sociocultural theory suggest that intercultural communication competence develops through participation in socially mediated activity rather than through isolated instruction (Idris & Widyantoro, 2019). Learning is understood as a process of becoming attuned to the norms and expectations that shape interaction, which are often implicit and context dependent. Within digital environments, this process is further shaped by the affordances of communication technologies, including their multimodal and asynchronous features. Competence does not emerge automatically through exposure to interaction. Pedagogical mediation plays a critical role in directing learners’ attention to relevant aspects of their communicative behavior and supporting its refinement over time (Shadiev et al., 2024b). This perspective underpins RQ3, which focuses on how technology-supported teaching designs contribute to competence development.

**Figure 1:**
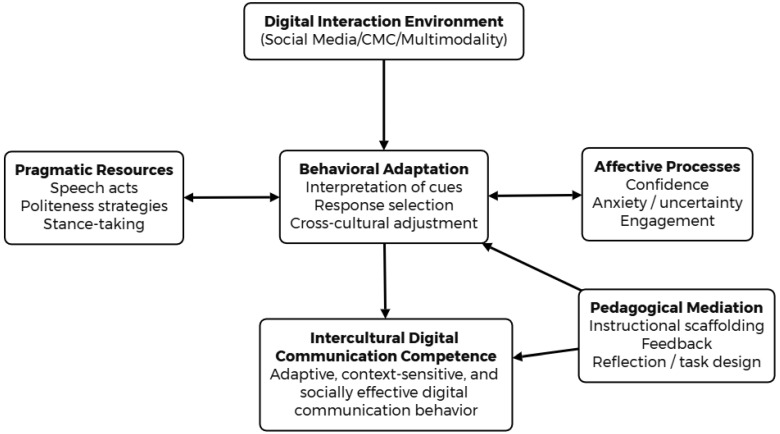
A behavioral model of intercultural digital communication competence development.

As shown in Figure 1, the framework conceptualizes intercultural digital communication competence as an emergent outcome of interaction in digitally mediated environments. Communication is shaped by the interplay of pragmatic resources, behavioral adaptation, and affective processes, while pedagogical mediation supports the development of adaptive and context-sensitive communicative behavior. This integrated framework provides the conceptual basis for the present review and guides the analysis of how communication is enacted and supported in higher education settings.

While prior research has often conceptualized social media as a tool or communication medium, this study approaches it as an interactional environment in which communicative competence develops through ongoing participation. This shift in perspective is theoretically meaningful. Viewing social media as a tool tends to emphasize its functional role in facilitating communication, whereas treating it as an environment foregrounds the dynamic, emergent, and socially situated nature of interaction (Kwayu, 2021). In such environments, meaning is negotiated through repeated exchanges, where learners adapt their communicative behavior in response to audience expectations and cultural differences. This perspective extends existing models of intercultural competence by emphasizing behavioral adaptation, multimodal meaning-making, and affective engagement as integral to competence development in digitally mediated contexts.

## 3. Methodology

This study adopted a systematic literature review approach to examine empirical research on cross-cultural pragmatics in social media-based communication among higher education students. The review focused on how intercultural digital communication competence is conceptualized and examined in digitally mediated interaction. To ensure transparency and replicability, the review was conducted in accordance with the PRISMA 2020 guidelines, which provide a structured framework for study identification, screening, and synthesis (Page et al., 2021).

### 3.1. Review Design and Protocol

The purpose of the systematic review was to map existing research, identify dominant themes and methodological tendencies, and highlight gaps in current scholarship on digitally mediated intercultural communication in higher education. Given the interdisciplinary nature of the topic, the review draws on studies from applied linguistics, communication studies, the learning sciences, and educational technology.

A review protocol was established before the literature search to guide the review process. This protocol specified the review objectives and research questions, outlined the search strategy, and defined explicit inclusion and exclusion criteria. Procedures for data extraction and synthesis were also determined in advance, helping to enhance consistency across studies and reduce the risk of selection bias.

### 3.2. Data Sources and Search Strategy

A comprehensive literature search was conducted across major academic databases to capture relevant research across disciplines. While additional databases, including ERIC and PsycINFO, are commonly used in educational and psychological research, this study prioritized Scopus and Web of Science for their broad interdisciplinary coverage and strong indexing of peer-reviewed journals in applied linguistics, communication, and digital education. This approach was intended to capture research at the intersection of cross-cultural pragmatics and digitally mediated interaction, which formed the core focus of this review. However, the exclusion of other databases may have limited the scope of the search, and future reviews could expand database coverage to further enhance comprehensiveness.

The search was limited to studies published between 2015 and 2025, a period corresponding to the increasing integration of social media and computer-mediated communication in higher education. Only studies published in English were included to allow for consistent analysis and comparison across sources.

Search strings were developed iteratively to balance coverage and specificity. Four key concept groups guided the search strategy, as summarized in Table 1: (1) social media and digital communication platforms, (2) cross-cultural and intercultural contexts, (3) pragmatics- and politeness-related features, and (4) higher education student populations. These concept groups were combined using Boolean operators to identify studies addressing the intersection of digital interaction, pragmatic practices, and intercultural communication in higher education settings.

### 3.3. Inclusion and Exclusion Criteria

To ensure relevance and consistency, explicit inclusion and exclusion criteria were applied during both the initial screening and full-text review stages (see Table 2). Studies were included if they focused on undergraduate or postgraduate students in higher education and examined communication in social media or other computer-mediated environments, such as social networking sites or messaging platforms. Eligible studies addressed cross-cultural or intercultural dimensions of communication and analyzed pragmatic or interactional features, including politeness strategies, speech acts, facework, emoji use, or other forms of multimodal meaning-making. Only empirical studies were considered, including those employing qualitative, quantitative, mixed-methods, or corpus-based designs. In addition, studies were required to be published in peer-reviewed journals or reputable conference proceedings.

Studies were excluded if they focused exclusively on non-university populations, such as children or general social media users, or if they discussed social media use without examining communicative practices in interaction. Research centered on digital literacy, technology adoption, or platform use without an intercultural or pragmatic focus was also excluded. Purely theoretical or conceptual work without empirical data was not considered, nor were studies published in languages other than English.

### 3.4. Study Selection and Screening Process

**Figure 2:**
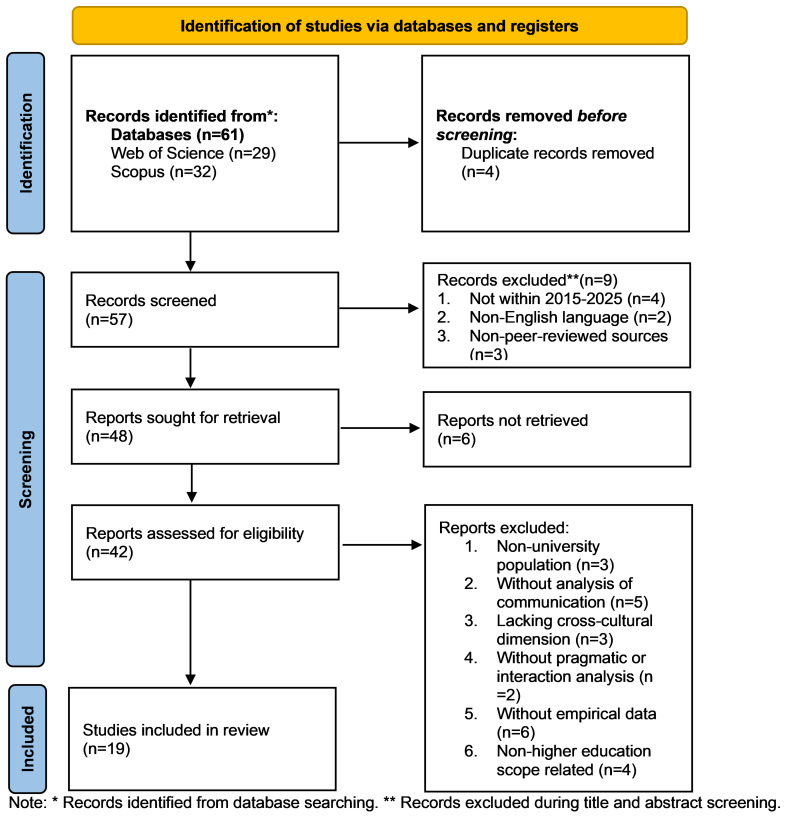
A PRISMA diagram of literature selection for review.

The study selection process followed the PRISMA 2020 framework to ensure transparency and consistency in identifying relevant literature. As illustrated in Figure 2, a total of 61 records were initially retrieved from two databases, including 32 from Scopus and 29 from Web of Science. Before screening, four duplicate records were removed, resulting in 57 records for initial screening.

During the first stage, we reviewed titles and abstracts to exclude studies that did not meet the basic inclusion criteria. At this stage, nine records were excluded, including studies published outside the 2015–2025 time frame (*n* = 4), non-English publications (*n* = 2), and non-peer-reviewed sources (*n* = 3). This process resulted in 48 reports being considered for full-text retrieval, of which six could not be accessed.

The remaining 42 full-text articles were then assessed for eligibility against the predefined inclusion and exclusion criteria. This stage involved a more detailed evaluation of the study focus, population, and methodological approach. We excluded a total of 23 articles for target reasons, including a focus on non-university populations (*n* = 3), lack of analysis of communication practices (*n* = 5), absence of a cross-cultural dimension (*n* = 3), insufficient attention to pragmatic or interactional features (*n* = 2), lack of empirical data (*n* = 6), and limited relevance to higher education contexts (*n* = 4).

Following the strict screening process, 19 studies were retained for inclusion in the final review. These studies met all inclusion criteria and formed the basis for the subsequent analysis. The PRISMA staged filtering process helped ensure that the selected studies were both relevant to the research focus and methodologically appropriate for examining intercultural digital communication competence in social media-based higher education contexts.

### 3.5. Quality Assessment

The Mixed Methods Appraisal Tool (MMAT) Version 2018 is a validated instrument for assessing the methodological quality of qualitative, quantitative, and mixed-methods studies. It should be noted that the MMAT assesses methodological quality at the level of individual studies, including research design, sampling strategy, data collection, and coherence of analysis (Hong et al., 2018). In this review, these criteria are used to inform a domain-based interpretation of risk of bias, as reflected in the traffic-light summary. However, the MMAT does not directly assess broader forms of bias (publication bias or cultural bias), which are considered separately in the interpretation of findings.

Due to the heterogeneity of the included studies in terms of research design, data type, outcome measures, and analytical approaches, a formal effect size comparison was not considered appropriate. The dataset includes qualitative, corpus-based, and mixed-methods studies, which do not provide comparable quantitative indicators for statistical synthesis. Instead, the review adopts a qualitative synthesis approach, supported by the MMAT to assess methodological quality.

To enhance objectivity, a double-review process was implemented. Two reviewers independently assessed the quality of each included study using the relevant MMAT criteria based on study design. The reviewers evaluated aspects such as the appropriateness of the research design, the clarity of data collection procedures, the rigor of analysis, and the coherence between data and interpretation. Any discrepancies between the two reviewers were resolved through discussion and consensus. In cases where agreement could not be immediately reached, a third reviewer was consulted to provide additional input and ensure consistency in the final evaluation (Gosak et al., 2021). To assess inter-rater reliability, Cohen’s Kappa coefficient was calculated. The resulting value of κ = 0.84 indicates a high level of agreement between the reviewers, suggesting that the quality assessment process was reliable and consistent.

Following MMAT guidance, studies were not excluded based solely on quality scores. Instead, the assessment was used to support the interpretation of the findings and to ensure that the included studies met an acceptable level of methodological rigor (Taylor & Hignett, 2014). The results of the quality assessment are summarized in Table 3.

The overall methodological quality of the included studies provides an important basis for interpreting the findings of this review. Most studies met a high proportion of the MMAT criteria, particularly in terms of clarity of research design and coherence between data and analysis. This suggests that the patterns identified in the review are grounded in studies with generally sound methodological foundations. Meanwhile, some variation in quality was observed, particularly regarding reporting detail and analytical depth, in a smaller number of studies. This variation reflects the diversity of research approaches in this interdisciplinary field. Considering these differences alongside the quality assessment, the review can provide a more balanced interpretation of the evidence.

**Figure 3:**
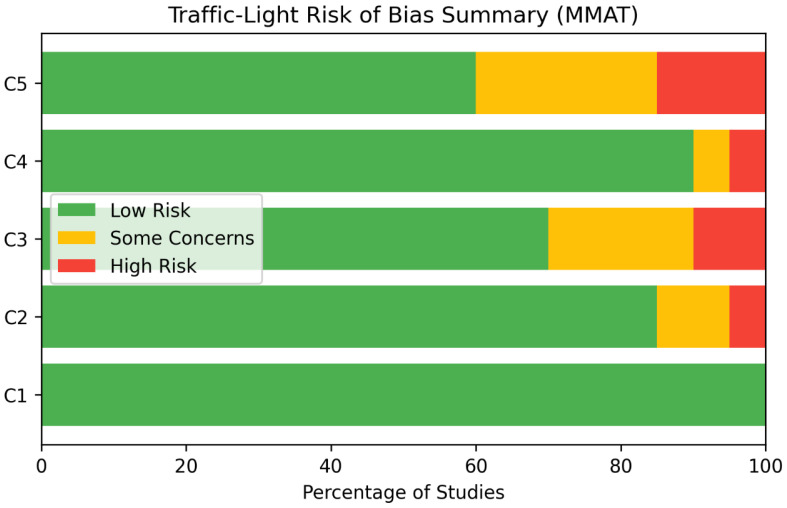
Traffic-light risk of bias summary based on MMAT criteria.

Figure 3 presents the proportion of studies meeting each MMAT criterion. Green indicates low risk (criterion met), yellow indicates some concerns (partially met or unclear), and red indicates high risk (criterion not met). While most studies demonstrate low risk across core domains, some variability is observed, particularly in criteria related to analytical depth and methodological integration.

## 4. Results

This section presents and interprets the findings of the systematic literature review based on the 19 studies included in the final dataset. The analysis is structured around the three research questions and is organized into two parts. The first part provides a descriptive overview of research trends and methodological orientations, while the second part develops a thematic synthesis that explains how intercultural digital communication competence is enacted and shaped in social media-based interaction.

### 4.1. Descriptive Overview of Research Trends (RQ1–RQ3)

Based on the research questions described above, this section presents a descriptive overview of the 19 studies included in the review. The section draws on Table 4, and it outlines publication trends, research contexts, digital platforms, and educational outcomes. This overview addresses the first research question, and it provides the empirical basis for the thematic analysis presented in Section 4.2.

#### 4.1.1. Research Development and Disciplinary Orientation (RQ1)

Table 4 shows that the included studies were published between 2015 and 2025. The number of studies increased after 2021, and this change suggests growing interest in digital interaction and intercultural communication. This interest also reflects the wider use of online and hybrid learning during this period (Alghasab & Alvarez-Ayure, 2023; Ismailov, 2021). Several studies treat social media and computer-mediated communication as teaching tools. These studies do not view them as secondary tools. They examine how these platforms support pragmatic development and intercultural learning (Zhang, 2021, 2023, 2024). This trend appears to reflect the growing reliance on digital media communication in higher education, a reliance that is becoming more pronounced against the backdrop of expanding online and blended learning environments. Across disciplines, there is a shift from treating social media and computer-mediated communication (CMC) as tools to viewing them as environments in which communicative behavior is shaped and negotiated.

Many studies come from applied linguistics and language education, but a large group uses educational technology and learning design perspectives. These studies connect closely to the aims of STEM education. In these fields, communication often takes place through digital collaboration platforms (Ghorbanzadeh & Sharbatiyan, 2024; Pikhart & Klímová, 2020). This also suggests that research is increasingly concerned with how individuals act and respond within digital environments, rather than only what linguistic forms they use.

#### 4.1.2. Cultural Contexts and Interactional Settings (RQ1)

The reviewed studies show strong representation from higher education settings in Asia. Many studies focus on China, Japan, and other Asian countries. Some studies also involve cross-border exchanges with Europe and Latin America, as shown in Table 5. Several studies focus on Chinese university students and examine pragmatic transfer, responses to compliments, and request strategies. They often use platforms such as WeChat and text-based computer-mediated communication (Li, 2023; Zhang, 2021, 2025).

Some studies use clear intercultural research designs, including online exchanges between students from Kuwait and Colombia. The interaction between Taiwanese and Japanese students is also involved. Other studies examine intercultural engagement in Hong Kong universities using ethnographic approaches (Alghasab & Alvarez-Ayure, 2023; Diaz et al., 2020; Fuchs, 2020). These intercultural settings reflect common conditions in global education. In these settings, students work in virtual teams, and they are required to manage different cultural norms in communication.

#### 4.1.3. Participant Characteristics and Educational Settings (RQ1)

As shown in Table 4, most participants across the 19 reviewed studies are undergraduate students, although sample sizes vary considerably. Some studies are small-scale qualitative investigations involving one or a few participants, allowing for close analysis of individual pragmatic development and identity work in digital interaction (Li, 2023; Sato, 2020). In contrast, larger experimental and quasi-experimental studies include several hundred learners and are typically designed to examine the effects of instructional interventions across broader populations (Zhang, 2021, 2023, 2024).

While many participants are enrolled in language-related programs, their interaction frequently takes place in technology-supported learning environments, such as inquiry-based online exchanges, telecollaborative projects, and game-mediated communication (Ismailov, 2021; Zhang, 2023). These settings share structural similarities with learning environments in STEM education, where students routinely coordinate tasks, solve problems, and negotiate roles through digitally mediated platforms.

#### 4.1.4. Digital Platforms and Communication Modalities (RQ1 & RQ3)

The reviewed studies examine a wide range of digital platforms (see Table 4), including instant messaging applications such as WeChat and WhatsApp, social networking sites such as Facebook, video-based collaboration tools, and game-based communication environments. Several studies foreground the role of multimodality, analyzing how emojis, stance markers, and emotional cues contribute to meaning-making in interaction (Diaz et al., 2020; Kousar et al., 2020; Liang, 2024).

For example, some studies examine emoji use in WhatsApp exchanges to show how affective and relational meanings are conveyed alongside text (Kousar et al., 2020), while others analyze stance softening in Taiwanese–Japanese computer-mediated interaction (Diaz et al., 2020). Emotional signaling in game-based environments has also been shown to shape engagement and interactional alignment (Zhang, 2023). Beyond platform-specific analyses, several studies demonstrate that culturally patterned discourse styles influence how messages are interpreted, even in text-based communication lacking explicit pragmatic markers. Experimental research on email communication, for instance, shows that culturally shaped writing styles affect readers’ judgments of politeness, willingness to engage, and sense of alignment with interlocutors (Hansen et al., 2015). These findings show the value of pragmatic awareness in digital channels that often appear neutral. Such insights are particularly relevant to STEM contexts, where communication frequently relies on brief, multimodal messages in shared digital environments, including project management systems and code repositories.

#### 4.1.5. Methodological Approaches and Analytical Focus (RQ1)

Most of the reviewed studies adopt qualitative or mixed-methods designs, combining discourse analysis with surveys, interviews, or task-based assessments (Alghasab & Alvarez-Ayure, 2023; Zhang, 2021). A smaller number of studies employ experimental or quasi-experimental approaches, often to examine the effects of pedagogical interventions. These studies compare unsupported computer-mediated communication with scaffolded interaction, telecollaborative work with face-to-face activities, or inquiry-based tasks with unassisted online exchanges (Ismailov, 2021; Zhang, 2021, 2024).

Such designs allow researchers to make more explicit connections between instructional arrangements and changes in pragmatic and intercultural outcomes. This methodological orientation is particularly relevant for understanding how teaching design can shape interactional practices in digitally mediated learning environments.

#### 4.1.6. Outcome Measures and Competence Dimensions (RQ2 & RQ3)

Across the 19 studies, a broad range of outcome measures is employed (see Table 4), extending beyond linguistic accuracy to include pragmatic appropriateness, strategy use, intercultural communication competence, learner engagement, and affective dimensions such as confidence and reduced anxiety. Several intervention studies report improvements in pragmatic performance following structured digital instruction (Zhang, 2021, 2023, 2024), while others document shifts in intercultural awareness and learner identity over time (Alghasab & Alvarez-Ayure, 2023; Ismailov, 2021; Sato, 2020).

These findings suggest that intercultural digital communication competence involves multiple interrelated dimensions, including behavioral flexibility, pragmatic awareness, and emotional readiness. This multidimensional view directly informs RQ2 and RQ3, highlighting the complexity of competence development in digitally mediated environments.

### 4.2. Thematic Synthesis (RQ2 & RQ3)

Based on a comparative analysis of the 19 studies summarized in Table 6, the review identified four interrelated themes that describe how intercultural digital communication competence is represented across the reviewed literature. These themes highlight recurring patterns in how competence is enacted and interpreted within digitally mediated interaction.

#### 4.2.1. Social Media as Environments for Behavioral Adaptation (RQ2)

Across the reviewed studies, social media and CMC environments are frequently described as spaces in which communicative behavior is shaped through participation. Learners appear to engage in processes of meaning negotiation, response adjustment, and interactional refinement over time, rather than simply applying predefined rules. Several studies reveal that intercultural pragmatic competence is closely associated with participation in ongoing interaction, where learners interpret contextual cues related to politeness, stance, and relational alignment (Alghasab & Alvarez-Ayure, 2023; Fuchs, 2020; Ismailov, 2021; Liang, 2024). From this perspective, competence is less often treated as a pre-existing skill, and it is more often described as an outcome of engagement with interactional contexts. While the environment of these processes leading to sustained development varies depending on the research, these environments may offer opportunities for feedback, adjustment, and experimentation.

#### 4.2.2. Micro-Pragmatic and Multimodal Features as Behavioral Resources (RQ2)

The theme highlights the role of micro-pragmatic and multimodal features as central resources in digital interaction. Across studies, elements involving stance markers, mitigation strategies, emojis, and short evaluative expressions are consistently reported as influencing how meaning is constructed and interpreted (Diaz et al., 2020; Kousar et al., 2020; Liang, 2024; Zhang, 2023).

These features have different effects in different contexts. across contexts. They appear to be shaped by culturally patterned expectations, which may result in variation in interpretation. Several studies point to instances where shared linguistic forms do not produce shared meaning, highlighting the potential for ambiguity and misalignment in intercultural communication (Tariq et al., 2020). This suggests that micro-level communication cues are key venues for cross-cultural understanding and negotiation, but they can also lead to misunderstandings.

#### 4.2.3. Instructional Support as a Mechanism for Behavioral Change (RQ3)

The third theme suggests that instructional design is frequently associated with differences in reported learning outcomes. Compared to studies that rely on unstructured interactions, research incorporating structured teaching elements (guided interaction, feedback, and reflective tasks) tends to report a more consistent pattern of pragmatism and cross-cultural awareness (Alghasab & Alvarez-Ayure, 2023; Ismailov, 2021; Zhang, 2021, 2024). These findings reveal that participation alone may not be sufficient to support competence development. On the contrary, learning appears to be more strongly linked to how interaction is structured and supported. However, given the diversity of research designs, these patterns may better be interpreted as associations, not causal effects.

#### 4.2.4. Affective Processes and Engagement in Interaction (RQ3)

The final theme focuses on affective dimensions, covering confidence, anxiety, motivation, and engagement (Ismailov, 2021; Lang-Rigal & Galarreta-Aima, 2019; Yoshida, 2020; Zhang, 2023). Across studies, these factors are frequently discussed in relation to participation and willingness to engage in interaction. Certain digital environments, like video-based or game-supported settings, are often associated with more positive affective experiences (Lang-Rigal & Galarreta-Aima, 2019; Zhang, 2023). However, the reliability of these environments in producing such outcomes is contingent upon the specific context. These findings indicate that affective processes are closely linked to communicative behavior, but may vary depending on interactional and contextual conditions.

In Summary, the four themes indicate that intercultural digital communication competence is described in the literature as involving multiple interacting dimensions. Across studies, competence is associated with the use of micro-pragmatic resources, participation in interaction, the presence of instructional support, and affective engagement. These elements appear to operate together, but their relative influence varies across contexts. This synthesis suggests that social media interaction alone does not guarantee competence development. It depends on how individuals interpret communicative cues, structured interactions, and adapt their behavior over time. In this regard, competence is better understood as a context-sensitive and interactionally negotiated process.

## 5. Discussion

This systematic literature review explored how intercultural digital communication competence appears and develops through social media-based interaction in higher education. The findings of this review suggest that intercultural digital communication competence is better understood as an adaptive, contextualized communication behavior, rather than a fixed set of linguistic or cultural skills. Across the reviewed studies, competence consistently emerges through ongoing engagement in digitally mediated interactions, in which individuals adjust their responses and negotiate meaning with others. This perspective highlights the importance of micro-pragmatic features, multimodal resources, and affective processes as integral components of competence. Thus, intercultural digital communication competence develops through participation and adaptation within communicative environments, not just through the acquisition of decontextualized knowledge alone.

### 5.1. Intercultural Digital Communication as Situated and Adaptive Behavior

The findings of this review suggest that intercultural digital communication competence is more appropriately understood as an interactionally situated and adaptive form of communicative behavior. Learners develop this competence through participation in real digital interaction, particularly in social media and telecollaborative contexts, where meaning, politeness, and relational alignment are continuously negotiated across cultural differences (Alghasab & Alvarez-Ayure, 2023; Fuchs, 2020; Ismailov, 2021; Liang, 2024). This perspective shifts the focus from what learners “possess” to how they “respond” within specific communicative contexts. The reviewed studies reveal that communicative behavior is shaped by ongoing interaction, contextual sensitivity, and cultural expectations.

### 5.2. Micro-Pragmatics and Multimodal Features as Behavioral Signals

A key implication of the findings is that intercultural understanding in digital environments is frequently negotiated through small and often ambiguous communicative cues. Across studies, stance markers, mitigation strategies, emojis, and short evaluative responses are shown to play a crucial role in expressing affect, managing face, and maintaining relational stability in intercultural communication (Diaz et al., 2020; Kousar et al., 2020; Liang, 2024; Zhang, 2023). Despite their importance, such features receive limited attention in many formal curricula.

As a result, similar expressions may be interpreted differently across contexts, which introduces a degree of uncertainty into intercultural interaction (Tariq et al., 2020). This reveals that competence involves the ability to use such features and recognize their variability and potential ambiguity. From this point of view, digital communication may increase both opportunities for alignment and risks of misunderstanding (Alzahrani & Elshemy, 2023; Grondin et al., 2019).

### 5.3. The Role of Pedagogical Mediation in Shaping Behavior

The findings also indicate that participation in digitally mediated interaction itself is not sufficient to support competence development. While interaction provides opportunities for engagement, the reviewed studies suggest that learning outcomes are more consistently reported in contexts where interaction is supported by pedagogical mediation (Alghasab & Alvarez-Ayure, 2023; Ismailov, 2021; Zhang, 2021, 2024).

This raises important questions about the assumption that exposure to authentic communication environments automatically leads to learning. Instead, the evidence suggests that competence development may depend on whether learners are supported in noticing, interpreting, and reflecting on communicative practices. From this perspective, pedagogical design also shapes how interaction is understood and used as a learning resource (Shadiev et al., 2024a; Wei, 2024).

### 5.4. Affective Processes and Participation in Intercultural Interaction

A further implication of the review is that affective factors appear closely linked to participation in intercultural digital interaction. Confidence, anxiety, motivation, and engagement are frequently discussed in relation to learners’ willingness to participate, take communicative risks, and sustain interaction (Ismailov, 2021; Lang-Rigal & Galarreta-Aima, 2019; Yoshida, 2020; Zhang, 2023). This is consistent with broader research suggesting that affective conditions influence participation and effort in communicative tasks (Najafi et al., 2024). However, these findings also point to potential inequalities in participation. Differences in language proficiency, cultural familiarity, and digital experience may influence who participates more actively and whose contributions are more visible. Thus, digital environments may serve as contexts where participation is shaped by both individual and structural factors (Gorokhovskaya, 2024; Hosseini et al., 2021; Malyndra et al., 2020). This reveals that intercultural digital communication competence involves adaptation and the ability to navigate uncertainty in interaction.

### 5.5. Technology, Feedback, and the Future of Communication Training

The findings also point to the potential role of emerging technologies in supporting communication awareness. Advances in learning analytics and AI-supported systems offer new possibilities for providing feedback on interactional features involved in tone, stance, and mitigation (Ghorbanzadeh & Sharbatiyan, 2024; Pikhart & Klímová, 2020). This perspective aligns with emerging research on learning analytics, natural language processing, and AI-supported feedback in education (Ma & Yang, 2025; Xia et al., 2024). From this standpoint, AI-supported systems may offer formative feedback on pragmatic features such as tone, stance, and mitigation, supporting learner reflection during or after interaction (Chan & Zary, 2019; Mosly, 2024). Such tools may be especially valuable in large international programs, where individualized feedback on communication is difficult to provide (Butow & Hoque, 2020; Mukadam et al., 2025).

At the same time, reliance on technological solutions raises questions about standardization and context sensitivity. Given the diversity of cross-cultural communication practices, the extent to which automated systems can interpret cultural nuances and the complexities of interactions remains unclear (Fountoulakis, 2024).

### 5.6. Limitations and Tensions in Digital Intercultural Interaction

Although the reviewed studies highlight the potential of social media and computer-mediated environments to support intercultural communication, several limitations and tensions are revealed. Digitally mediated interaction may also increase the likelihood of misunderstanding in contexts where communicative cues are reduced and interpreted differently across cultures. The use of brief and multimodal expressions also increases the rate of ambiguity and misaligned interpretations. Another typical phenomenon is that participation in online environments does not occur under equal conditions. Differences in language proficiency, digital literacy, and cultural familiarity may shape who participates more actively and whose contributions are more visible or valued. Therefore, social media environments may facilitate communication and reproduce existing inequalities. These considerations suggest that intercultural digital communication competence involves the ability to adapt effectively and navigate uncertain and potential miscommunication in digitally mediated contexts.

### 5.7. Geographical Distribution and Generalizability of Findings

An additional point that merits consideration is the geographical distribution of the studies included in this review. A substantial proportion of the research has been conducted in Asian contexts. While these studies provide valuable insights into intercultural digital communication, this concentration may also shape the patterns identified in the analysis. Communication practices observed in these contexts may reflect culturally specific norms related to politeness and group orientation that do not fully generalize to other cultural environments. Therefore, in interpreting the findings of this review, we identified this geographical bias and set a base for future research to broaden its geographical scope in order to better capture the diversity of intercultural communication practices in the context of digital higher education.

While this review emphasizes the central role of micro-pragmatic and multimodal features in shaping intercultural digital communication, these findings can also be examined within broader dimensions of digital media interaction. These dimensions include the close relationship between identity and self-presentation issues and pragmatic choices, as individuals utilize language and multimodal resources to construct and present their social identities in intercultural contexts (Wilmot et al., 2023). Furthermore, power relations can influence the choice and interpretation of communication strategies, given the differences in language proficiency, cultural background, social status, and emotional sensitivity among digital media users (Roshid & Chowdhury, 2023). The unique features offered by digital platforms, generally characterized by persistence and multimodality, also play a significant role in shaping how communication unfolds, influencing the form and interpretation of interactions. Considering these dimensions in conjunction with micro-level features allows for a more comprehensive understanding of intercultural digital communication as a process of social embedding and technological media.

## 6. Conclusions

### 6.1. Summary of Key Findings

Four key insights emerge from the review. Firstly, research on cross-cultural pragmatics in social media–mediated communication is methodologically diverse but shows several recurring patterns. And many studies are situated in higher education contexts in Asia and the Global South.

Further, the findings indicate that intercultural digital communication competence is closely associated with pragmatic and interactional processes, including speech acts, stance-taking, politeness strategies, and multimodal resources. These features appear to play an important role in meaning-making, while also introducing potential for ambiguity and misinterpretation.

Moreover, the review highlights that participation in digital interaction alone may not be sufficient for competence development. More consistent outcomes are reported in contexts where interaction is supported by pedagogical design.

Finally, affective factors are frequently linked to participation and communicative behavior, although these relationships appear to vary across contexts.

### 6.2. Implications for Higher Education and Existing Models

These findings carry important implications for higher education, where digitally mediated collaboration is increasingly central. As global teamwork becomes a routine feature of academic and professional practice, intercultural digital communication competence can no longer be treated as a secondary or implicit outcome. Instead, it may be recognized as an important component of academic and professional preparation (Andrienko et al., 2021; Basantes-Andrade et al., 2025).

These findings also have implications for how existing models of intercultural competence are conceptualized. Traditional frameworks often emphasize knowledge, attitudes, and skills as relatively stable components. However, this review reveals that such models may underrepresent the dynamic and context-dependent nature of communication in digital environments. Researchers need to pay greater attention to behavioral adaptation, multimodal meaning-making, and affective engagement as core dimensions of competence. This shift moves the focus from competence as a static attribute to competence as an emergent process shaped through interaction. Therefore, models of intercultural communication may need to be extended to explain the role of the digital environment as a venue for the continuous negotiation and refinement of communication behaviors.

### 6.3. Directions for Future Research

Several avenues for future research follow from this review. Further empirical studies are needed to examine how intercultural digital communication competence shapes teamwork, problem-solving, and professional identity development. Longitudinal and design-based research could offer deeper insight into how pragmatic competence evolves and how different forms of pedagogical support influence learning trajectories.

Furthermore, emerging technologies, including learning analytics, natural language processing, and AI-based feedback systems, present promising opportunities for studying and supporting communicative behavior at scale. Future research should also broaden its geographical and cultural scope, as many regions remain underrepresented in the current literature. Expanding this coverage is essential for developing models of intercultural digital communication competence that reflect the diversity of global higher education.

### 6.4. Concluding Remarks

This review offers a more specific account of intercultural digital communication competence than is typically presented in the literature. In addition to supporting the finding that competence is universally developed through interaction, the study further indicates that competence development is closely related to how learners interpret and respond to subtle and often ambiguous communication cues in digital media environments. Micro-pragmatic and multimodal features (tone, stance, and emoji) emerge as key sites where intercultural meaning is negotiated, but also where misunderstanding frequently occurs.

A further contribution of this review is to question the assumption that participation in social media interaction is inherently beneficial for competence development. Across the reviewed studies, interaction alone does not appear to guarantee learning outcomes. Conversely, development is more consistently associated with contexts in which learners are supported in observing, interpreting, and reflecting on communicative practices. This shows that the value of social media environments lies not simply in providing opportunities for interaction, but in deliberate structural design and purposeful pedagogical intervention.

The findings also reveal that intercultural digital communication involves navigating uncertainty and asymmetry in interaction. The competence was further engaged with managing ambiguity and responding to culturally variable interpretations. This perspective shifts attention from communication as a transferable skill to communication as an adaptive and context-dependent process.

Since most related research focuses on specific geographical contexts, the more significant implication of this review lies in identifying recurring patterns that may inspire future research. The findings also highlight the need for deeper investigation into how learners identify and adapt to micro-level communication differences across diverse digital and cultural environments.

## Figures and Tables

**Table 1:** Search Strings with Field Restrictions Used in Databases.

Database	Search String
Scopus	TITLE-ABS-KEY (“social media” OR Instagram OR Facebook OR Twitter OR TikTok OR WeChat OR “computer-mediated communication” OR “digital communication”) AND TITLE-ABS-KEY (intercultural OR “cross-cultural” OR multicultural OR “second language” OR L2) AND TITLE-ABS-KEY (pragmatic* OR politeness OR “speech act*” OR compliment* OR “face work”) AND TITLE-ABS-KEY (“higher education” OR universit* OR “college student*” OR undergraduat* OR postgraduat*)
Web of Science (Core Collection)	TS = (“social media” OR Instagram OR Facebook OR Twitter OR TikTok OR WeChat OR “computer-mediated communication” OR “digital communication”) AND TS = (intercultural OR “cross-cultural” OR multicultural OR “second language” OR L2) AND TS = (pragmatic* OR politeness OR “speech act*” OR compliment* OR “face work”) AND TS = (“higher education” OR universit* OR “college student*” OR undergraduat* OR postgraduat*)

Note: * represents a truncation symbol used in database search strategies to retrieve multiple word variations.

**Table 2:** Summary of Inclusion and Exclusion Criteria.

Criteria	Inclusion	Exclusion
Population	Focused on higher education students.	Non-university populations
Context	Examined communication occurring in social media or computer-mediated environments	Without an analysis of communication
Cultural Scope	Addressed cross-cultural or intercultural dimensions of communication	Lacking a cross-cultural dimension
Focus of Analysis	Investigated pragmatic or interactional features	Without pragmatic or interactional analysis
Study Design	Reported empirical findings	Without empirical data
Educational Scope	Related to higher education or learning contexts	Non-higher education related
Publication Type	Peer-reviewed sources	Non-peer-reviewed sources
Language	English	Non-English

**Table 3:** Quality Assessment of Included Studies Using MMAT.

Study (Author, Year)	Study Type	Criteria Met (Out of 5)	Quality Level
(Fuchs, 2020)	Qualitative	5/5	High
(Zhang, 2021)	Quantitative	5/5	High
(Liang, 2024)	Qualitative	4/5	High
(Hansen et al., 2015)	Quantitative	5/5	High
(Tariq et al., 2020)	Quantitative	3/5	Medium
(Pikhart & Klímová, 2020)	Quantitative	3/5	Medium
(Yoshida, 2020)	Qualitative	4/5	High
(Lang-Rigal & Galarreta-Aima, 2019)	Quantitative	3/5	Medium
(Li, 2023)	Qualitative	4/5	High
(Diaz et al., 2020)	Qualitative	5/5	High
(Alghasab & Alvarez-Ayure, 2023)	Mixed Methods	5/5	High
(Ismailov, 2021)	Quantitative	4/5	High
(Zhang, 2025)	Quantitative	5/5	High
(Sato, 2020)	Qualitative	4/5	High
(Zhang, 2023)	Quantitative	5/5	High
(Zhang, 2024)	Quantitative	5/5	High
(Kousar et al., 2020)	Qualitative	3/5	Medium
(Ghorbanzadeh & Sharbatiyan, 2024)	Quantitative	3/5	Medium
(Gutiérrez-Santiuste & Ritacco-Real, 2023)	Mixed Methods	4/5	High

Note: Quality levels were determined based on MMAT criteria: High (4–5 criteria met), Medium (2–3 criteria met). No studies were excluded based on quality, in line with MMAT recommendations.

**Table 4:** Characteristics of Included Studies.

Articles	Platform/Technology	Pragmatic Focus	Educational Outcomes
(Fuchs, 2020)	Facebook	Intercultural engagement, discourse norms	Informal learning of intercultural and pragmatic awareness
(Zhang, 2021)	Text-based CMC, data-driven instruction	Compliment responses	Improved pragmatic appropriateness & strategy diversity
(Liang, 2024)	Facebook discussions	(Dis)agreement, emotional expressiveness	Enhanced stance awareness and relational alignment
(Hansen et al., 2015)	Email-based CMC	Politeness cues, style alignment	Cultural cues influenced perception & willingness to interact
(Tariq et al., 2020)	Facebook	Pragmatic ambiguity, interpretation	Raised awareness of pragmatic misinterpretation online
(Pikhart & Klímová, 2020)	AI-enhanced eLearning systems	Digital interaction expectations	Highlighted the need for intelligent communication support
(Yoshida, 2020)	Text chat	Socio-affective scaffolding	Improved engagement and interaction quality
(Lang-Rigal & Galarreta-Aima, 2019)	Video-based virtual exchange	Oral interaction, confidence	Reduced anxiety and increased speaking confidence
(Li, 2023)	WeChat	Request strategies, pragmatic transfer	Revealed culturally influenced pragmatic patterns
(Diaz et al., 2020)	Text-based CMC	Stance-taking, mitigation	Improved awareness of pragmatic softening
(Alghasab & Alvarez-Ayure, 2023)	Telecollaborative discussions	Intercultural competence	Pedagogical mentoring enhanced intercultural interaction
(Ismailov, 2021)	Inquiry-based virtual exchange	Intra- and intercultural communication	Increased engagement and intercultural confidence
(Zhang, 2025)	Online interaction tasks	Bidirectional pragmatic transfer	Demonstrated language influence in digital contexts
(Sato, 2020)	Social media, interviews	Identity and subjectivity	Supported reflective intercultural awareness
(Zhang, 2023)	Messaging tools	Compliment responses in gaming	Game-mediated interaction supported pragmatic development
(Zhang, 2024)	Telecollaborative video vs. face-to-face	Compliment responses	outperformed face-to-face practice
(Kousar et al., 2020)	WhatsApp	Emoji use and pragmatic meaning	Emojis functioned as culturally situated pragmatic cues
(Ghorbanzadeh & Sharbatiyan, 2024)	University websites, social platforms	Engagement and interaction value	Platform features shaped communicative engagement
(Gutiérrez-Santiuste & Ritacco-Real, 2023)	Videoconferencing/telecollaboration	Intercultural communication behavior	Development of behavioral, and cognitive dimensions of intercultural competence

**Table 5:** Distribution of Included Studies.

Included Articles	Distribution of the Study Population
(Gutiérrez-Santiuste & Ritacco-Real, 2023)	Spain
(Zhang, 2025)	China
(Sato, 2020)	Japan
(Zhang, 2023)	China
(Zhang, 2024)	China
(Kousar et al., 2020)	Pakistan
(Ghorbanzadeh & Sharbatiyan, 2024)	Iran
(Alghasab & Alvarez-Ayure, 2023)	Kuwait VS. Colombia
(Ismailov, 2021)	Japan
(Fuchs, 2020)	China
(Zhang, 2021)	China
(Liang, 2024)	International
(Hansen et al., 2015)	International
(Tariq et al., 2020)	International
(Pikhart & Klímová, 2020)	International
(Yoshida, 2020)	Japan
(Lang-Rigal & Galarreta-Aima, 2019)	International
(Li, 2023)	China
(Diaz et al., 2020)	International

**Table 6:** Thematic Synthesis of Included Studies.

Theme	Core Focus	Representative Studies
Theme 1	Social Media as Environments for Behavioral Adaptation	(Fuchs, 2020), (Liang, 2024), (Diaz et al., 2020), (Alghasab & Alvarez-Ayure, 2023), (Ismailov, 2021), (Sato, 2020), (Ghorbanzadeh & Sharbatiyan, 2024)
Theme 2	Micro-Pragmatic and Multimodal Features	(Liang, 2024), (Li, 2023), (Diaz et al., 2020), (Zhang, 2023), (Zhang, 2024), (Kousar et al., 2020), (Zhang, 2025)
Theme 3	Instructional Support for Behavioral Change	(Zhang, 2021), (Alghasab & Alvarez-Ayure, 2023), (Ismailov, 2021), (Zhang, 2023), (Zhang, 2024), (Ghorbanzadeh & Sharbatiyan, 2024), (Gutiérrez-Santiuste & Ritacco-Real, 2023)
Theme 4	Affective Processes and Engagement in Interaction	(Yoshida, 2020), (Lang-Rigal & Galarreta-Aima, 2019), (Ismailov, 2021), (Zhang, 2023), (Zhang, 2024)

## Data Availability

The original contributions presented in this study are included in the article. Further inquiries can be directed to the corresponding author.
